# The Effectiveness of Virtual Reality Rehabilitation in Patients with Knee and Hip Osteoarthritis

**DOI:** 10.3390/jcm9082639

**Published:** 2020-08-14

**Authors:** Joanna Byra, Krzysztof Czernicki

**Affiliations:** 1 Faculty of Health Sciences, Jagiellonian University Medical College, Michalowskiego 12, 31-126 Cracow, Poland; 2 Department of Rehabilitation, Lesser Poland Orthopaedic and Rehabilitation Hospital, Modrzewiowa 22, 30-224 Cracow, Poland; kczernicki@gmail.com

**Keywords:** rehabilitation, virtual reality, knee osteoarthritis, hip osteoarthritis, knee arthroplasty, hip arthroplasty, review

## Abstract

Osteoarthritis (OA) is a common health problem leading to pain, limitation in physical function, a decrease in the quality of life and disability. OA affects 60–70% of the population above 65 years of age all over the world, and is associated with a high cost of healthcare. The main method of treatment of OA, apart from pharmacotherapy and surgery, is comprehensive rehabilitation. Advances in medical technology have resulted in the possibility of using computer-assisted interventions in rehabilitation. The present narrative review is aimed at investigating the effectiveness of virtual reality (VR) in the rehabilitation of elderly patients with knee or hip osteoarthritis, including patients after arthroplasty. This literature review based on Preferred Reporting Items for Systematic Reviews and Meta-Analyses (PRISMA) guidelines was carried out in five databases: PubMed, Medline, Web of Science, Scopus and PEDro. It includes ten randomized controlled trials focused on the application of games and biofeedback in the rehabilitation of patients with knee and hip osteoarthritis. There are no conclusive reports that interventions based on VR are more effective than standard physical therapy. Moreover, evidence regarding patients after total hip arthroplasty (THA) is very scarce. The effectiveness of VR-based rehabilitation is unclear, although interventions based on VR are promising in view of pain management, postural and proprioception training. However, this evidence is not sufficient to create clinical guidelines and further high-quality studies are needed.

## 1. Introduction

Degenerative joint disease or osteoarthrosis (OA) is a progressive joint disease characterized by the focal loss of cartilage, little evidence of the typical form of inflammation, and by the hypertrophy of adjacent bone and soft tissue. Such a definition is synonymous with the hypertrophic form of chronic (osteo)arthritis [1,2].

OA is a well known world-wide cause of a progressive disability [3]. OA is increasing in prevalence across the world mainly due to the aging population [4]. OA affects approximately 70% of women and 60% of men older than 65 years [4]. However, the estimated prevalence and incidence of OA may vary depending on the definition of the disease, the joints considered and the population being studied [3].

As a degenerative disease, OA increases with age, exacerbating the associated social and medical problems, leading in the long run to an extensive need of holistic treatment. The risk of OA increases considerably every decade after the age of about 45 years [5].

Osteoarthritis progressively degrades the patient’s independence, mobility and participation in social life, thus reducing the overall quality of life [5]. Dominant clinical problems related to OA include pain, limited joint mobility, decreased muscular strength, impaired proprioception and increased incidence of falls. OA also affects mental health, resulting primarily in depressive episodes [5,6].

Non-pharmacological methods of treatment of degenerative joint disease are aimed at pain control, improvement of function and the quality of life of the patient, with the goal of minimizing the adverse effects of the therapy [7,8,9].

Over the course of the recent decades, arthroplasty or joint replacement surgery became the principal method of orthopaedic surgical treatment of advanced degenerative joint disease, primarily in the case of the hip joint and the knee joint. However, the currently available total hip arthroplasty (THA) and total knee arthroplasty (TKA) indication criteria are based on limited evidence [10].

Rehabilitation, especially work-related rehabilitation (physical therapy) is widely employed as an element of complex therapy or as a standalone intervention during the treatment of degenerative joint disease. Kinesiotherapy on a regular basis and habitual physical activity are substantial in the prevention of OA and in managing the initial stages of the disease. Preoperative rehabilitation treatment, as a preparation for joint surgery, shows benefits with regards to the length of hospital stay and discharge destination (home/rehabilitation ward) [11]. Postoperative rehabilitation treatment is desirable after surgery in view of decreased morbidity, increased satisfaction and safety after discharge from the surgical ward [12].

Rehabilitation is widely used following joint replacement operations, allowing the restoration of the function of the affected joint and preservation of obtained results. A progressive exercise program is beneficial considering recovery after THA/TKA, allowing for a faster recovery and an increase in the range of motion [13,14].

Recent advances in medical technology resulted in a gradual introduction of computer-assisted interventions into rehabilitation. In recent decades, new technologies in rehabilitation were developed from the simplest forms of biofeedback implementations into hardware platforms and motion capture systems augmented by biofeedback, augmented reality (AR) systems and virtual reality (VR) systems. The range of employed technologies varies from non-expensive, popular gaming platforms to highly specialized systems with dedicated hardware and software platforms. Nowadays, the threat of the SARS-CoV-2 pandemic, where physical interactions could be prohibited, may result in an increased interest in contactless methods of rehabilitation, a condition fulfilled by telerehabilitation [15].

The influence of interventions based on new technologies on the course of rehabilitation, particularly their hypothetical advantage over standard work-related rehabilitation interventions in OA, has not yet been verified. Therefore, the present study was conducted to determine, based on the current literature review, whether virtual reality rehabilitation significantly improves the physical function of elderly patients suffering from knee or hip osteoarthritis, including patients after arthroplasty. An additional purpose of this review was to assess the impact of this type of intervention on patients’ quality of life, adherence, acceptance, and its usefulness in the process of rehabilitation.

## 2. Materials and Methods

### 2.1. Literature Search Strategy

The methodology of this literature review was based on the Preferred Reporting Items for Systematic Reviews and Meta-Analyses (PRISMA) guidelines [16]. The review was conducted following the PRISMA checklist (Supplementary Materials; Table S1). The literature review was carried out in five databases: PubMed, Medline, PEDro, Web of Science and Scopus.

The primary search keywords were “hip replacement OR knee replacement OR osteoarthritis AND virtual reality AND rehabilitation” and their synonyms. For PubMed, we used the following search strategy: “(((((((hip osteoarthritis) OR knee osteoarthritis) OR knee replacement) OR hip replacement) OR hip arthroplasty) OR knee arthroplasty)) AND (((((exergam*) OR video game) OR virtual reality) OR augmented reality)) AND ((((((rehabilitation) OR physical therapy) OR physiotherapy) OR activity) OR exercise)) [All Fields]”.

The results of this review were based on dependent variables, which mainly concerned a detailed description of the functional state of the patient with osteoarthritis or after arthroplasty. The following variables were involved: physical functions based on the validated scales of a functional evaluation of the knee and hip, body balance, gait pattern, range of movement, muscle strength assessment, evaluation of pain level and proprioception. Due to the multidimensional effects of rehabilitation and the special type of intervention used, it was decided to evaluate the impact of VR on the quality of life, adherence and patient motivation, based on including studies.

The main aim of this literature review and search strategy outline were formulated based on the PICO(T) tool [17].

- Population—adult patients after total hip replacement OR total knee replacement OR knee osteoarthritis OR hip osteoarthritis;
- Intervention—virtual reality, exergames rehabilitation;
- Comparison—standard rehabilitation;
- Outcome—physical function, balance, gait, range of motion, muscle strength, pain, proprioception;
- Time—last 10 years (January 2010–April 2020);
- (Type of study)—randomized controlled trials.

### 2.2. Study Selection and Data Extraction

Initially, both authors created the criteria for eligibility. One researcher searched databases (last search: 28th April 2020). Then, the researchers selected abstracts and further full-text articles independently, using an automation tool—Rayyan [18]. The relevant data from the included studies were extracted separately by both researchers and later compared. Potential conflicts at every stage of study selection and data extraction were solved in discussion.

The articles were included using the following criteria: published in the last 10 years, randomized controlled trials, written in the English language, targeted to adults’ participants with knee/hip osteoarthritis or knee/hip arthroplasty, exergames or virtual reality exercises.

Studies that did not meet the above criteria were excluded. Additionally, unpublished studies, the protocols of randomized controlled trials and conference reports were also excluded. The review includes studies from the last 10 years due to the rapid development of new technologies and the need to obtain the most current results.

### 2.3. Quality of Included Studies

The methodological quality assessment of the included articles was made on an 11 point PEDro scale [19]. The PEDro scale is a reliable and correct scale for assessing the methodological quality of randomized clinical trials [20]. It is also one of the adequate scales for assessing the specifics of clinical trials in the field of physiotherapy and rehabilitation [19,21]. The results of the evaluation of five studies that were available in the PEDro database (https://www.pedro.org.au/) were extracted, whereas the remaining five studies were evaluated independently by two researchers, and the possible differences were solved through discussion. The following interpretation of the scale was used: 1–3 points low quality, 4–6 points moderate quality, above 7 points high quality.

## 3. Results

### 3.1. Searching Results

**Figure 1:**
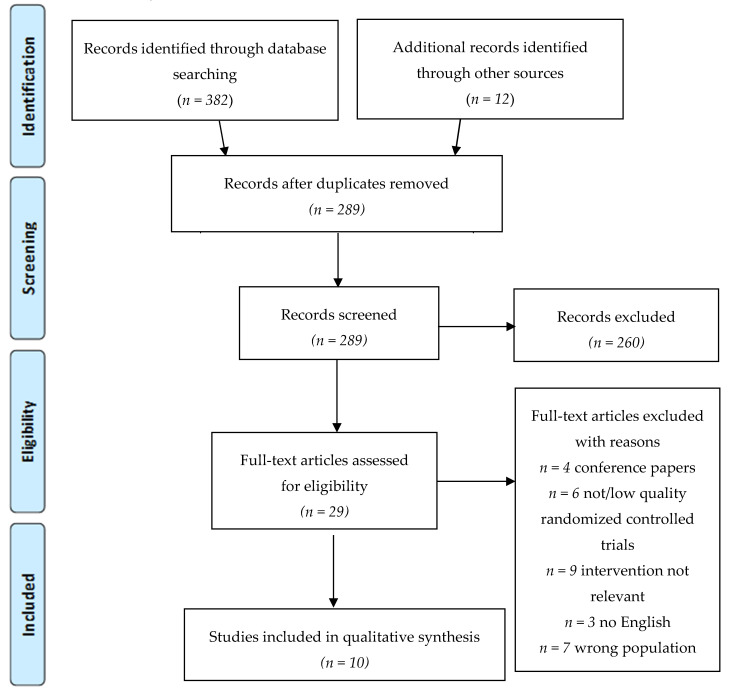
Flowchart of the article selection process (2009 Preferred Reporting Items for Systematic Reviews and Meta-Analyses (PRISMA) flow diagram).

A total of 394 articles was found, and after the removal of duplicates, 289 titles and abstracts were screened. From the screened abstracts, 29 studies were read in full text. Nineteen of those were excluded for the following reasons: conference papers, low quality randomized controlled trial (RCT) with insufficient data, intervention not relevant or wrong population (concerning also other types of OA, ankle arthroplasty, spondylarthritis or a not well defined population). The final ten randomized controlled trials were included for qualitative analysis. Figure 1 presents the PRISMA flowchart of the study selection.

### 3.2. Demographic Data

In the total 10 RCTs, there were 492 subjects with a mean age of 60.6 years. Nine of the trials included information regarding sex, and thus the percentage of male participants was 41.6%. The studies focused on the rehabilitation of the following orthopaedic conditions: seven total knee arthroplasty (TKA), two knee osteoarthritis (KOA), one total hip arthroplasty (THA). The authors decided to include one study [22] that compares two groups after knee surgery in view of the VR rehabilitation: patients after TKA and after anterior cruciate ligament (ACL) tear repair. It was considered desirable to include this study because of the valuable reports on the effectiveness of the VR training. In addition, in the study group, only two patients underwent ACL repair, and the remaining 15 subjects underwent TKA. It was recognized that the initial purpose of the intervention aimed solely at increasing the range of knee movement was the same for both conditions. Moreover, the specifics of rehabilitation aimed at increasing the range of knee movement are similar and should not affect the results of this study.

A detailed description of the studies included in the review is presented in Table 1.

### 3.3. Methodological Evaluation

The methodological quality of the analysed research rated on the PEDro scale ranged from moderate quality—4 points [22,29]—to high quality—8 points [24]—with a mean score of 5.9 points (moderate quality). Four studies were high quality above 7 points on the PEDro scale [22,23,27,28].

The most common type of bias was due to the lack of participants and therapists blinding (in all studies). In most physiotherapy research, blinding is difficult due to the specifics of carrying out interventions. However, blinding all test assessors is possible and should be used in RCT, which concerned about half of the included studies [23,24,26,28,30]. The highest quality in evidence-based medicine and evidence-based practice is obtained by randomized double-blind or triple-blind control trials. Blinding prevents bias caused by the expectations of the patients or therapists (performance bias) and in the assessment by assessors (detection bias) [32]. However, double or triple blinding in rehabilitation studies is often difficult or impossible because the patient and the therapist are aware of the intervention [33]. However, a single blinding in physical therapy is possible if the investigator does not have information about the intervention and the randomization of patients [34]. Some studies indicate that the absence of blinding affects the overestimation of the results [35] and the level of blinding of rehabilitation studies is poor [36].

Another limitation was the lack of the intention-to-treat analysis which concerned eight out of 10 included RCTs (it occurred only in [24,27]). The intention to treat is necessary to maintain a prognostic balance in the results between groups. Results of participants who did not complete the intervention but were initially assigned by randomization should be included in the analysis to maintain the similarity of the compared groups, which minimizes the risk of bias [37]. Missing data can be considered as treatment failure [38].

The main limitations of the included trials were the small sample size [22,24,26,28,29,30,31] and the lack of long-term follow up [22,24,27,28,29].

### 3.4. Type of Technology

#### 3.4.1. Exergames

Physical exercises in the form of exergames and virtual reality are increasingly used in rehabilitation. Among the included studies, eight described games and virtual reality training in patients after TKA and THA. Gianola et al. [23] used the Virtual Reality Rehabilitation System (VRRS, Khymeia, Italy) in rehabilitation after TKA. The VRRS is a device that allows the correct performance of exercises based on visual and auditory biofeedback and a system of interactive games focused on body balance.

Lin et al. [24] applied exercises based on interactive games using the Hot Plus system (Supreme Investment Co., Taipei, Taiwan). The patients with knee OA participated in the game by moving the legs and tilting the torso in each direction, transferring the weight of the body to the sensory pillows that they were standing on. Exercises were regulated at three levels of difficulty and visual and auditory biofeedback informed about their correct performance. The purpose of VR exercises was to increase the range of motion and muscular strength of lower extremities and to improve body balance and coordination. A similar type of game, aimed at increasing muscle strength, body balance and the leg range of motion was applied by Elshazly et al. [27]. The patients with knee OA played the Light Race game, consisting of balancing the body weight from one leg to the other in the right direction, to move on a virtual platform displayed on the TV screen.

Jin et al. [25] used virtual reality (Mide Technology Inc., Cangzhou, China) on the second day after surgery as a supplement to early rehabilitation in patients after TKA. The patients put on a virtual reality headset and had motion sensors connected to the operated limb. They performed exercises that consisted of playing a game simulating boat rowing, using active flexion and extension of the operated knee.

On the other hand, Lehrl et al. [31] used an interactive cognitive game, Kawashima’s Brain Training: How Old Is Your Brain? (Nintendo; Kyoto, Japan), for patients after THA. The goal was to improve the executive functions—memory length and information processing speed. The stimulation of cognitive functions had a positive effect on the physical state, as patients were more willing to perform the exercises.

Christiansen et al. [28] used The Nintendo Wii Fit Plus game associated with Wii Balance Board (Nintendo of America, Inc., Redmond, WA, USA) to aid the recovery of the operated lower limb weight-bearing symmetry in the home rehabilitation system. The patients played interactive games with the progression of the operated limb load and the level of difficulty ranging from static to dynamic exercises. They received visual and auditory biofeedback after correctly completing the task. Ficklscherer et al. [22] also applied the Nintendo Wii in the rehabilitation of patients after knee surgeries—TKA (*n =* 26) and ACL plastic surgery (*n =* 4). The aim of this study was to assess the usability and safety of this device, considering the age of the participants and their experience with modern technology. Fung et al. [30] also evaluated the effectiveness of the therapy on the function of the operated limb, body balance, mobility and muscle strength in people after TKA using the Nintendo Wii.

#### 3.4.2. Feedback

Koo et al. [26] studied the effectiveness of analgesic therapy using augmented reality, which was a combination of mirror therapy and virtual reality (enhanced reality using real-time image processing technique). The patients after TKA performed exercises based on visual biofeedback, in which the image of the operated leg’s movement was replaced by a visualization of the movement of the healthy leg. Another kind of visual feedback was applied by Ayoade et al. [29] as home physical therapy for patients after TKA. It consisted of a knee joint exercise system based on the use of motion sensors and real-time visual biofeedback (Rehabilitation Visualization System, RVS). The exercises were focused on strengthening the muscles and increasing the range of motion.

### 3.5. Effects of Intervention—Primary Outcomes

#### 3.5.1. Physical Function

The evaluation of the condition of the knee after TKA and with OA were conducted in eight out of 10 studies. Among the most commonly used scales [24,25,26,27] was the WOMAC scale (The Western Ontario and McMaster Universities Osteoarthritis Index), which includes the assessment of pain, stiffness, and physical functioning [39]. Two studies [23,25] showed a statistically greater improvement of knee condition in the VR training group than in the controls for patients after TKA. However, in one trial [23], improvement was obtained only in reducing joint stiffness. On the other hand, the authors [22,23,26,29,30] presented no major functional improvement in VR rehabilitation for TKA patients. For patients with knee OA, VR rehabilitation had good results for physical function [27] and for initial improvement in pain reduction [24].

In contrast, one of the included studies concerning THA [31] showed that the gaming group had a much better improvement in hip function than the control group in the Harris Hip Score (HHS). Despite the observed improvement in function and mobility, there was no statistically significant improvement in the second scale of hip function—Merle d’Aubigné score (PMA). However, the contrast in the statistical significance of the results of hip functional assessment in these two scales may be due to the variance in their scores, as the more detailed HHS has 0–100 point scale, and PMA, which is less detailed, has 0–18 point scale.

#### 3.5.2. Balance

Three included studies evaluated the impact of VR training on body balance. For this purpose, stabilometric platforms [23,24] or the Activity-Specific Balance Confidence Scale (ABCS) [30] were used. Two studies [23,30] showed no statistically significant improvement in the exergames training group for the patients after TKA. Additionally, Christiansen et al. In [28], the load on the operated limb after TKA was evaluated, using the peak vertical ground reaction force (vGRF) on a stabilometric platform during the Five Times Sit-to-Stand Test (FTSST). No better results were obtained in the study group. However, the speed of FTSST test performance was statistically better in the study group than in the control group.

On the other hand, one study [24] indicated a greater increase in dynamic stability in the group of knee OA patients that performed VR training.

#### 3.5.3. Gait

Four studies assessed the gait, using marching tests of varying duration: 2 min [30], 6 min [26], 10 min [24] and 12 min [28]. In addition, one study [28] assessed the gait pattern, including the mobility of the joints of the operated extremity. Only in one study [24] was an increase in the gait efficiency achieved in the group of VR training for the patients with knee OA. Three other studies [26,28,30] consisting of patients after TKA showed no improvement in gait performance.

#### 3.5.4. Range of Motion

Assessment of the range of motion of the operated joint after TKA using goniometer was performed in five studies [23,25,26,29,30]. In three trials [25,26,29], a statistically significant increase in the movements of the operated knee was obtained in the exergame groups. Furthermore, the patients increased the knee range of motion faster, as assessed by the time needed to reach 60 and 90 degrees of flexion [25]. In the control group, there were cases of deterioration of the motion during therapy, which was not noted in the experimental group [29]. In two studies [23,30], no statistically significant differences in the range of motion were noticed.

#### 3.5.5. Proprioception

Knee joint proprioception was evaluated for patients with knee OA [27] and after TKA [23] and in both studies, significant improvement was obtained in the group using VR training compared to the control group.

#### 3.5.6. Muscular Strength

The muscular strength of the operated leg was examined by a dynamometer [23] or by using the timed-stands test (TST) [26]. In both trials, there were no statistically significant differences in the improvement of muscle strength between the study groups and controls for the patients after TKA.

#### 3.5.7. Pain

The level of pain was assessed by five researchers using the following scales: Visual Analog Scale [23,25,26,27] and Numeric Pain Rating Scale [30]. In two trials [25,26] statistically significant greater pain reduction was demonstrated in the VR training group, as well as the analgesic effect related to the length of training [26] was longer for the patients after TKA. However, the other two studies [23,30] did not show such a difference for these patients, which in one of them [23] may be due to the initial disproportion in the results of the assessment of the level of pain between the test and control group. Only one study [27] assessed the level of pain in patients with knee OA and suggests improvement in favour of VR rehabilitation.

### 3.6. Effect of Intervention—Secondary Outcomes

#### 3.6.1. Quality of Life

The impact of VR training on increasing the quality of life was tested by various scales: EuroQoL five-dimensional (EQ-5D) [23], World Health Organization Quality of Life-Brief Vision (WHOQOL-BREF [24], short form survey from the SF-36 Health Survey (SF-12) [29] and health-related quality of life (HRQOL) [27]. Lin et al. [24] emphasized a significant improvement in the physical health domain of WHOQOL-BREF in the VR-training group compared to the control group for patients with knee OA. The beneficial effect of VR training on the quality of life for knee OA is proved also by Elshazly’s research [27]. In contrast, the other two studies [23,29] concerning patients after TKA did not show the impact of VR training on increasing the quality of life of patients.

#### 3.6.2. Adherence and Motivation

Four trials [22,24,29,30] focused on the acceptance and adaptation of patients for VR-based rehabilitation. Lin et al. [24] emphasized that the 4 week VR rehabilitation program had a 100% degree of adherence, and in the control group where standard exercises were performed, the rate was 93%. Ficklscherer et al. [22] payed attention to the lack of side effects, safety and acceptance of exergames as a form of physiotherapy. Fung et al. [30] stated that gaming rehabilitation is a satisfactory and acceptable form of treatment. However, Ayoade et al. [29] assessed the level of adherence and the involvement of patients in the rehabilitation process by assessing the number of days in which they participated in the planned exercises. The level of involvement in both groups was the same. In addition, the patients’ experiences regarding the type of home rehabilitation were assessed using the Intrinsic Motivation Inventory (IMI) questionnaire and there was no difference between the degree of interest and motivation between the VR rehabilitation and standard exercises.

#### 3.6.3. Inpatient/Outpatient/Home Physical Therapy

Seven trials [22,23,24,25,26,27,30] performed the intervention in a hospital or clinic assisted by a therapist/researcher, while two trials [28,29] carried out the intervention as a home rehabilitation program for patients after TKA. Christiansen et al. [28] offered the patients a therapist’s control 1–3 times a week and provided them with instructions on how to perform the exercises. Ayoade et al. [29] also assessed the effectiveness of exercises at home using a visual communication system with the therapist (video conferences). In addition, Ayoade et al. [29] assessed the usability of VR rehabilitation using the System Usability Scale (SUS) questionnaire and obtained results suggesting that home VR rehabilitation was associated with greater patient involvement, was motivating because it yielded information about the progress of exercises and improved communication between the therapist and the patient.

## 4. Discussion

The present review was aimed at assessing, based on the present-day literature, whether virtual reality significantly affects the process of rehabilitation of patients suffering from knee or hip osteoarthritis, including patients after knee or hip arthroplasty. The results of the review suggest that the effectiveness of VR-based rehabilitation varies. Among the included studies, five indicated a statistically significant improvement in the measured outcomes in the VR-group compared to the control group [24,25,26,27,31]. However, the others did not show a statistically significant difference in the improvement of the physical state compared to the controls [22,23,28,29,30]. The effectiveness of VR rehabilitation was observed in four studies in patients with knee osteoarthritis [24,25,26,27], and in one study in patients with hip osteoarthritis [31]. In addition, only one RCT study [31] concerning VR rehabilitation in patients after THA was included. Results of the current review suggest that the effectiveness of VR-based rehabilitation compared to standard physiotherapy is debatable. In others similar studies, the most promising effects of VR were observed for balance and proprioception [40,41]. Wang et al. [42] also noted very limited evidence for THA. Physical rehabilitation in knee or hip osteoarthritis and arthroplasty is focused on pain relief, gait re-education, the improvement of body balance, proprioception of limb joints, increment of the mobility, and the development of muscular strength [43].

Regarding primary outcomes, VR-based rehabilitation is promising in the field of pain relief [25,26,27]. This conclusion is supported by Lin et al. [40] and Wang et al. [42], but negated by Blasco et al. [41]. Pain perception is multidimensional, which is why VR rehabilitation can be used in the conservative treatment of pain based on a change in its experience. It was noticed that diverting attention from pain, creating an image of the illusion of a healthy limb while performing a task (game) allowed the patients to perform more difficult exercises [44]. The effect of mirror therapy and virtual reality on brain plasticity has been proven, and its effectiveness has so far been demonstrated in phantom pain therapy [45,46], in neuropathic pain therapy [47] and in patients after strokes [48,49,50]. The reduction of pain achieved using VR is a promising phenomenon that can contribute to reduction in the use of pharmacological agents and improves the quality of life of post-operative patients. The use of exergames in reducing pain and improving the quality of life has good results in women with fibromyalgia [51]. Cacciata et al. [52] found that the improvement of health-related quality of life in participants with high adherence to exergaming and those in a center-based setting appeared to have the most promising effects. Collado-Mateo et al. [53] emphasized that VR training may be useful in reducing musculoskeletal pain, but more detailed research is needed on specific disease entities.

Obtaining an adequate gait pattern and gait speed is an important goal of the rehabilitation of patients after knee or hip arthroplasty and in the prevention of falls in this group of patients. Training with the use of VR affects the improvement of these functions; however, its effectiveness in this area has been proven only in patients with knee OA [24]. However, it has been noted that training on VR stability platforms has contributed to improving mobility and gait rate in women with risk of falls [54] as well as in the elderly people [55].

Few reports [23,30] regarding the impact of VR rehabilitation on improving body balance in patients after TKA arthroplasty show no significant improvement compared to the traditional way of rehabilitation. However, stabilometric platforms using games for body balance exercises are currently widely used in rehabilitation [56]. Wii Board Balance has been proven to be a useful diagnostic tool for assessing body balance and selecting orthopedic aids in women after TKA [57] and for the evaluation of muscular strength of the lower limb in the elderly [58]. The effect of treadmill training with VR on improving body stability in children with spastic cerebral palsy has been demonstrated [59]. The effectiveness of VR training focused on body balance has been demonstrated in functional improvement in patients after strokes [60,61], with back pain [62], as well as in gait re-education and improvement of body balance in patients after TKA [63]. Blasco et al. [41] researched the efficacy of VR tools for total knee replacement and suggested that an augmented Virtual Reality Tools (VRT) physical therapy could be more effective for overcoming balance limitations than standard physical therapy.

Proprioception means the sense of the position of the body in space. Impairment in this area leads to knee instability as well as gait and balance dysfunctions [64]. Gianola et al. [23] and Elshazly et al. [27] were the only ones to study the improvement of proprioception in patients after TKA [23] and knee OA [27], obtaining significant relief in favor of VR. Other researchers assessed the effect of exergames training on knee proprioception in healthy older men [65], lower limb proprioception in people with risk of falling [66], and upper limb proprioception in stroke patients [67]. Therefore, this is a promising field for further research in the area of proprioception improvement, especially in patients after THA.

The improvement of the range of motion and muscle strength is an objective assessment of a knee function. Its mobility limitations are symptomatic of the progression of knee osteoarthritis, and the quick restoration of the range of motion after knee arthroplasty is necessary to regain physical function. VR training has been proven to increase the range of knee movement after TKA [25,26,30] much more than standard physical therapy. In the two cited studies [23,26], which assess the muscular strength of the lower limb after TKA surgery, in both cases no major impact of VR rehabilitation was demonstrated. Other authors’ reports noted the benefit of using VR to increase muscular strength in women with risk of falling [68], whereas Villafaina et al. [69] noted that the increase in muscular strength through exergames training obtained in women with fibromyalgia is greater but short-lived.

In one study [31], patients after THA improved their physical function after VR cognitive training. Physical activity and cognitive training are two factors that reduce the risk of dementia [70], which is associated with an increased risk of falls and leads to disability [71]. Mental exercises also had a positive effect on reducing pain perception [72]. It has been noticed that the combination of physical and cognitive training improves body balance and working memory in older people more than motor training alone [73].

Secondary outcomes of the included studies concerned adherence, motivation, and usability. The feasibility of VR rehabilitation depends on patients’ adherence to modern technology, and the simplicity of general use should be maintained, especially for out-patient application. Economic efficacy comes in view regarding, among other factors, a reduction of qualified human resources needed.

Results of the present review suggest that VR rehabilitation has been assessed with high adherence [24], as a safe and acceptable procedure without side effects [22], providing satisfaction for participants [30] and the motivation to exercise [29]. Moreover, Molina et al. [74] found a consensus between studies in the positive motivational aspect of the exergames in older adults.

An extremely important element of the rehabilitation process is its continuation at home. Virtual reality and modern technology mean that this task should be carried out quickly, easily, and safely, and under the therapist’s control. Nowadays, especially in the times of isolation due to the SARS-CoV-2 pandemic, limited access to rehabilitation has forced a modification of the stationary treatment process into treatment using recent technology (telerehabilitation). Only two included studies [28,29] used VR as a part of home rehabilitation for patients after TKA. Gonzalez-Franco et al. [75] tested the rehabilitation protocol for patients after TKA in a group of healthy volunteers as a part of home rehabilitation using modern technology. Participants emphasize the possibility of intuitive and accessible teaching of the correct performance of exercises. Piqueras et al. [76] emphasized that the use of telerehabilitation is as effective in improving knee function after TKA as traditional home rehabilitation. It is a promising method of rehabilitation, bringing quick results compared to the group performing exercises alone, motivating because of the contact with the therapist, reducing the costs associated with getting to the hospital.

The present review has some limitations. Only randomized controlled trials published in the last 10 years in English were included. The reason was the choice of high-quality RCT, providing reliable information on the effectiveness of the used intervention. The protocol of a randomized controlled trial avoids bias and is considered the gold standard in clinical trials [77]. The subject of this review was limited to orthopedic rehabilitation in osteoarthritis and TKA/THA, as so far, the focus has been placed on the use of VR in neurological rehabilitation [78], rehabilitation after amputations [45,46], and in the treatment of pain [47].

At present, a small, but systematically increasing number of studies regarding virtual reality in orthopedic rehabilitation requires verification of its quality and effectiveness. Thanks to this, it would be possible to create guidelines that allow the safe incorporation of VR into the rehabilitation of patients with osteoarthritis.

The fundamental dilemma related to studies regarding the area of rehabilitation and physical therapy is associated with the well defined research protocol, with the particular emphasis on the coherent selection of the study group, incorporation the control group into the study, the proper selection of the control group, the sufficient size of the samples, and adequate measurement tools. The question of blinding persists as well and was discussed elsewhere. Further studies are needed, but these studies should strictly follow protocols founded on evidence-based medicine guidance, with the emphasis on appropriate research protocol.

## 5. Conclusions

There is no conclusive evidence that interventions based on virtual reality are more effective than standard physiotherapy treatment in the rehabilitation of patients suffering from osteoarthritis, including patients after total knee arthroplasty. Evidence regarding patients after total hip arthroplasty is very scarce. Interventions based on virtual reality are promising in view of pain management, range of motion, and proprioception.

Future research studies, notably randomized controlled trials, with a well defined research protocol, are needed to determine the effectiveness of virtual reality in the rehabilitation of osteoarthritis, regarding rehabilitation after total hip arthroplasty.

## Figures and Tables

**Table 1:** Characteristics of the included studies.

No	First Author, Year, Country	PEDro Scale	Main Objective	Participants	Intervention/Technology	Outcomes	Research Tools	Main Results
**1**	Gianola et al. [23] 2020, Italy	7/10	The effectiveness of VR rehabilitation vs. standard rehabilitation for physical function after primary TKA	Adults 45–80 years old 3–4 days after primary unilateral TKA: all *n* = 74 study *n* = 35 controls *n* = 39	Sixty minute daily training for at least 5 days All participants performed passive ROM exercises on Kinetec knee continuous passive motion system and functional exercises. Study—VR games focused on balance, proprioception and function of lower limbs Controls—standard postoperative physiotherapy	Pain; knee disability quality of life; patients’ assessment of their condition; functional performance; leg muscle strength; knee ROM; proprioception; balance; medication assumption	VAS WOMAC EQ-5D GPE FIM dynamometer goniometer stabilometric platform VRRS	No significant difference in pain reduction and other outcomes, except improved proprioception in the study VR group.
**2**	Lin et al. [24] 2020, Taiwan	8/10	A comparison of VR games exercises and standard physical exercises in patients with knee OA	Patients aged 40–85 with knee OA (Kellgren and Lawrence Score ≥ 2): all *n =* 80 study *n =* 40 controls *n =* 40	Three times a week for 4 weeks with a follow-up All received 20 min of hot packs and 20 min of TENS Study—active video games using the Hot Plus system focused on muscle strength, coordination and ROM of limbs Controls—standard exercises	knee disability; quality of life; psychosocial distress; fatigue; pain; work ability; balance	WOMAC WHOQOL-BREF HADS MFI CPG WAI Biodex Stability System	No significant difference between VR training and standard exercises in improving knee disability, but games improved dynamic balance, physical functional performance, and physical health more than therapeutic exercises.
**3**	Jin et al. [25] 2018, China	5/10	The effects and benefits of VR training in postoperative patients after TKA	Patients after primary unilateral TKA: all *n =* 66 study *n =* 33 controls *n =* 33	Standard therapeutic exercises + 30 min three times a day: Study—VR training (Mide Technology) from the second day of therapy, focus on knee flexion (rowing boat) Controls—3 sets of active knee flexion exercises	knee disability; evaluation of early results of TKA; pain; ROM	WOMAC HSS VAS goniometer	VR training effects better at improving knee functional recovery, ROM and relieving pain after TKA than standard exercises.
**4**	Koo et al. [26] 2018, South Korea	6/10	The effectiveness of enhanced reality on analgesia and physical function after TKA	Patients with unilateral TKA: all *n =* 42 study *n =* 22 controls *n =* 20	Enhanced reality analgesia visual biofeedback (combination of the VR, real-time motion capture, mirror therapy using real-time image processing technique) Study—intervention was provided shortly after physiotherapy for five times a week for 2 weeks Controls—intervention was provided for five times a week for 1 week	Pain; ROM; knee disability; endurance and aerobic capacity; lower extremities; strength; medication assumption;	VAS goniometer WOMAC 6MWT TST	Analgesia and improvement in ROM in both groups were achieved, but in the study group that lasted longer than in controls.
**5**	Ficklscherer et al. [22] 2016, Germany	4/10	The assessment of the Nintendo Wii as an appropriate and safe tool in rehabilitation after orthopedic knee surgery	Patients with TKA or anterior cruciate ligament (ACL) tear: all *n =* 30 study *n =* 17 controls = 13	Four weeks of intervention Study—exergames on The Nintendo Wii and standard physical therapy Controls—standard physical therapy	Knee function and disability	IKDC MCKRS TLKS	No significant difference between VR training and standard exercises in improving knee disability, but slightly greater improvement in the Wii group. Intervention without a negative influence on patients.
**6**	Elshazly et al. [27] 2016, Saudi Arabia	7/10	A comparison of the effectiveness of VR training over sensory motor training in the treatment of osteoarthritis	Patients with chronic OA (> 3 mths) WOMAC: 71 points: all *n =* 60 virtual reality training *n =* 20 Qsensory motor training *n =* 20 controls *n =* 20	Three times per week for 8 weeks Study—virtual reality training Light Race VR interactive game 15-30′ ControlsI—sensory motor progressive training II—conventional exercise training warm-up, walking, cool-down	Pain intensity; joint proprioception; knee disability; quality of life	VAS perception sense WOMAC HRQOL	Significant improvement in the outcome measures in all the training methods. VR training showed a substantial improvement over the other methods.
**7**	Christiansen et al. [28] 2015, USA	7/10	The effectiveness of weight-bearing (WB) biofeedback training on WB symmetry and functional joint moments following unilateral total knee arthroplasty	Patients with unilateral TKA: all *n =* 26 study *n =* 13 controls *n =* 13	Study—weight-bearing biofeedback on the Nintendo Wii Fit Plus and Wii Balance Board and physical therapy daily for 6 weeks Controls—physical therapy twice per day for 6 weeks	Gait/walking speed; lower limb; weight-bearing ratios (WBRs); lower limb joint movement	12 m walkway—gait speed FTSST	No significant difference between VR training and controls in WBR. FTSST time improved in the study group compared to the control group. The tendency for improved walking speed in the study group at 26 weeks (*P* = 0.068).
**8**	Ayoade et al. [29] 2014, Canada	4/10	The presentation of rehabilitation visualization system (RVS); the assessment of usability and feasibility of the RVS at home	Patients with TKA in the early phase of post-operative rehabilitation: all *n =* 21 study *n =* 11 controls *n =* 10	Ten days of training in the hospital and then at home for up to 6 weeks Study—rehabilitation visualization system and exercise handbook Controls—exercise handbook only	Knee ROM; functional performance; health surveys; rehabilitation experience; usability	Goniometer OKS SF-12 survey IMI SUS	Overall, no significant difference between RVS training and controls. However, RVS made home rehabilitation more engaging and improved the communication between patients and the therapist.
**9**	Fung et al. [30] 2012, Canada	5/10	The examination of the Nintendo Wii Fit as an acceptable adjunct to physiotherapy concerning balance, ROM, muscle strength and function in outpatients following TKA	Outpatients after TKA: all *n =* 50 study *n =* 27 controls *n =* 23	Twice a week for 75 min Study—15 min of exergames focused on postural control and balance in The Nintendo Wii Fit and 60 min of physical therapy Controls—60 min of physical therapy and 15 min of lower extremity strengthening and balance training	Balance; knee ROM; postural control; lower leg function; pain	2MWT Goniometer ABCS LFES NPRS satisfaction survey	No significant difference between study and controls. Wii Fit is potentially acceptable as an adjunct to physical therapy intervention in view of balance, postural control and use of the lower extremities.
**10**	Lehrl et al. [31] 2012, Germany	5/10	The enhancing of rehabilitation using mental activation	Patients after THA: all *n =* 32 study *n =* 16 controls *n =* 16	Study—30 min per day for 12 days of video game Dr. Kawashima’s Brain Training: How Old Is Your Brain? Controls—without intervention	Hip function and disability	HHS PMA	Significant improvement in hip function obtained in the study group in HHS, but not in PMA.

Abbreviations: VR—virtual reality, TKA—total knee arthroplasty, THA—total hip arthroplasty, ROM—range of motion, VAS—Visual Analogue Scale, WOMAC—Western Ontario and McMaster Universities osteoarthritis index, EQ-5D EuroQol five-dimensional questionnaire, GPE—the global perceived effect score, FIM—functional independence measure questionnaire, VRRS—Virtual Reality Rehabilitation System, OA – osteoarthritis, TENS—Transcutaneous Electrical Nerve Stimulation, WHOQOL-BREF—World Health Organization Quality of Life-Brief Vision, HADS—Hospital Anxiety and Depression Scale, MFI—Multidimensional Fatigue Inventory, CPGQ—Chronic Pain Grade Questionnaire, WAI—Work Ability Index, HSS—Hospital for Special Surgery knee score, 6MWT—6 min walk test, TST—timed-stands test, IKDC—International Knee Documentation Committee score, mCKRS—Modified Cincinnati Knee Rating System, TLKS—Tegner Lysholm Knee Score, HRQOL—health-related quality of life, FTSST—Five Times Sit-to-Stand Test, OKS—Oxford knee score, SF-12 survey—short form survey from the SF-36 Health Survey, IMI—Intrinsic Motivation Inventory, SUS—system usability scale, 2MWT—2 min walk test, ABCS—Activity-Specific Balance Confidence Scale, LFES—Lower Extremity Functional Scale, NPRS—Numeric Pain Rating Scale, HHS—Harris Hip Score, PMA—Merle d’Aubigné score.

## Supplementary Materials

The following are available online at https://www.mdpi.com/2077-0383/9/8/2639/s1, Table S1: PRISMA checklist.
